# The Anthelmintic Activity of *Nepeta racemosa* Lam. Against Gastrointestinal Nematodes of Sheep: Rosmarinic Acid Quantification and In Silico Tubulin-Binding Studies

**DOI:** 10.3390/pathogens14010077

**Published:** 2025-01-15

**Authors:** Büşra Karpuz Ağören, Mahmut Sinan Erez, Esma Kozan, Aydın Dağyaran, Mevlüt Akdağ, Eduardo Sobarzo-Sánchez, Esra Küpeli Akkol

**Affiliations:** 1 Department of Pharmacognosy, Faculty of Pharmacy, Başkent University, Ankara 06810, Turkey; busrakarpuz@baskent.edu.tr; 2 Department of Parasitology, Faculty of Veterinary Medicine, Afyon Kocatepe University, Afyonkarahisar 03200, Turkey; mserez@aku.edu.tr (M.S.E.); esmakozan@aku.edu.tr (E.K.); 3 Çay Directorate of Agriculture and Forestry, Çay, Afyonkarahisar 03706, Turkey; aydin.dagyaran@tarimorman.gov.tr; 4 Department of Pharmaceutical Chemistry, Faculty of Pharmacy, Afyonkarahisar Health Sciences University, Afyonkarahisar 03030, Turkey; mevlut.akdag@afsu.edu.tr; 5 Centro de Investigación en Ingeniería de Materiales, Facultad de Ciencias de la Salud, Universidad Central de Chile, Santiago 8370292, Chile; 6 Department of Organic Chemistry, Faculty of Pharmacy, University of Santiago de Compostela, 15782 Santiago de Compostela, Spain; 7 Department of Pharmacognosy, Faculty of Pharmacy, Gazi University, Ankara 06330, Turkey

**Keywords:** *Nepeta racemosa*, Lamiaceae, anthelmintic, rosmarinic acid, in silico, tubulin, HPLC

## Abstract

Gastrointestinal nematodes (GINs) inflict significant economic losses on sheep and goat farming globally due to reduced productivity and the development of anthelmintic resistance. Sustainable control strategies are urgently needed including the exploration of medicinal plants as safer alternatives to chemical anthelmintics. This genus of plants is used for anti-inflammatory, antioxidant, and antimicrobial activities. In this study, we aimed to evaluate the anthelmintic activities of *Nepeta racemosa* Lam. MeOH extract, *n*-hexane, dichloromethane (DCM), ethyl acetate (EtOAc), *n*-buthanol (*n*-BuOH) and aqueous (H_2_O) subextracts, and quantify rosmarinic acid in the active extract by the HPLC method, and perform in silico molecular docking studies of rosmarinic acid to examine its binding interactions with tubulin. The anthelmintic activity of the plant extracts on gastrointestinal nematode eggs and larvae (L3) of the sheep was assessed using in vitro test methods such as the egg hatch assay and larval motility assay, conducted over a 24 h period (1, 2, 3, 4, 6, 8, 24). All extracts exhibited 100% effectiveness in the egg hatch inhibition assay, regardless of concentration (50–1.5625 mg/mL). The EtOAc subextract shows the highest effectiveness at 79.66%, followed by the MeOH extract at 74.00%, water at 64.00%, *n*-hexane at 67.00%, and DCM at 61.00%, and the lowest effectiveness is observed with *n*-BuOH at 51.66% in the larval motility assay. The major compound of EtOAc extract, the most active extract of *N. racemosa*, was determined as rosmarinic acid and its amount in the extract was determined as 14.50 mg/100 mg dry extract. The amount of rosmarinic acid in the MeOH extract was found to be 0.21 mg/100 mg dry extract. *n*-Hexane, DCM, *n*-BuOH, and H_2_O extracts’ rosmarinic acid content was lower than the LOQ value. As tubulin plays an important role in the mechanism of anthelmintics, the major compound of the most active extract (NR-EtOAc) rosmarinic acid was docked onto the colchicine-binding site of the tubulin (5OV7) protein. Rosmarinic acid showed a similar activity spectrum to the anthelmintic drug albendazole. The discovery of low-cost and low-toxicity anthelmintic compounds is very important.

## 1. Introduction

Gastrointestinal nematodes (GINs) are a major parasitic threat to grazing ruminants, particularly sheep and goats, causing substantial economic losses globally. Key genera include *Haemonchus*, *Trichostrongylus*, *Teladorsagia*, *Chabertia*, *Cooperia*, *Nematodirus*, and *Oesophagostomum*. Infections range from subclinical weight loss and reduced productivity to severe health problems, including diarrhea, anemia, and potentially death [1,2,3]. The widespread use of anthelmintic drugs has led to the development of anthelmintic resistance (AR), diminishing the effectiveness of many treatments and contributing to significant economic losses. The estimated annual cost of AR in Europe alone is EUR 38 million and rising [4], underscoring the urgent need for alternative control methods.

This necessitates a multifaceted approach encompassing genetic selection, improved pasture management, optimized nutrition, biological control (e.g., nematophagous fungi), vaccine development, and herbal remedies. The overuse of chemical anthelmintics also raises concerns regarding residues in animal products and environmental impact [1,5]. The development of resistance to multiple anthelmintic classes, including benzimidazoles, macrocyclic lactones, imidazothiazoles, and newer drugs like monepantel, emphasizes the need for integrated strategies combining rational drug use with alternative approaches. The growing demand for food and the potential for GINs to increase greenhouse gas emissions further highlight the critical importance of developing sustainable parasite control solutions. The exploration of medicinal plants as safer, cheaper alternatives to chemical drugs holds significant promise [6,7].

The *Nepeta* genus, one of the largest within the Lamiaceae family (comprising 236 genera and 6900–7200 species), includes approximately 280 species distributed across central and southern Europe, western, central, and southern Asia, and North Africa. A particularly high concentration of *Nepeta* diversity is found in southwestern Asia, notably Türkiye and Iran, establishing this region as a hotspot for the genus. Türkiye alone boasts 33 recorded *Nepeta* species, 17 of which are endemic. Species of the *Nepeta* genus (Lamiaceae) are rich in bioactive compounds such as phenolic compounds, nepetalactones, and essential oils, which contribute to their various medicinal uses. Traditionally, *Nepeta* species have been used in folk medicine for their anti-inflammatory, febrifuge, antimicrobial, antiseptic, antispasmodic, anti-asthmatic, antitussive, diuretic, and digestive benefits [8]. In Türkiye, their traditional uses extend to treating colds, cancers, coughs, rheumatism, wounds, obesity, and stomach ailments [9,10]. The anthelmintic potential of *Nepeta* species has been evaluated through in vitro and in vivo studies on nematodes and other parasitic worms. In a study on the anthelmintic activity of methanol extract of *Nepeta cataria*, it was reported that it showed significant in vitro anthelmintic activity against gastrointestinal nematodes of sheep [11]. In another study, ethanol, methanol, acetone, and water extracts of *Nepeta cadmea* (an endemic species from Türkiye) showed dose-dependent anthelmintic activity [12]. There is no study on the anthelmintic effect mechanism of *Nepeta* species.

Microtubules are essential for cell division (as the major constituents of the mitotic spindle), the cytoskeleton (as constituents of the cilia and flagella), intracellular transport, signal transduction, and motility. Because microtubules are essential for multiple cellular processes, they are excellent options for inhibiting cell division, growth, and proliferation [13]. The broad class of anthelmintics known as benzimidazoles, like albendazole, possess a high affinity for the *β*-tubulin protein and function by attaching to the protein’s colchicine-binding site [14].

Considering this literature information, this study aimed to evaluate the anthelmintic activities of the different polarity extracts prepared from *Nepeta racemosa* [methanol (MeOH) extract and *n*-hexane, dichloromethane (DCM), ethylacetate (EtOAc), *n*-butanol (*n*-BuOH), and aqueous (H_2_O) subextracts] through the egg hatch assay and larval motility assay, quantification of the major compound/s in the active extract by the HPLC method, and in silico molecular docking studies of rosmarinic acid to examine its binding interactions with tubulin.

## 2. Results and Discussion

In this study, the anthelmintic activities of *Nepeta racemosa* (NR) (MeOH extract, *n*-hexane, DCM, EtOAc, *n*-BuOH, and H_2_O subextracts) depending on the concentration were evaluated. All extracts exhibited 100% effectiveness and were found to be statistically significant compared to control groups in the egg hatch inhibition assay, regardless of concentration (50–1.5625 mg/mL) or time interval (1–24 h). The statistical significance of each mortality (%) result was compared with the control groups (PBS and DMSO). Increased nematocidal activity against gastrointestinal nematodes of sheep larvae was observed with increasing extract concentrations in the larval motility assay.

The efficacy of the *N. racemosa* extract and subextracts (at a concentration of 50 mg/mL at the 24th hour) with different solvents, in descending order, is as follows: the ethyl acetate subextract (NR-EtOAc) shows the highest effectiveness of 79.66%, followed by 74.00% for methanol extract (NR-MeOH), water (NR-H_2_O) at 64.00%, 67.00% for *n*-hexane (NR-*n*-hexane), and 61.00% for dichloromethane (NR-DCM). The lowest effectiveness of 51.66% was registered for *n*-butanol (NR-*n*-BuOH). The distribution over time of the effects of different concentrations of *N. recomosa* on gastrointestinal nematodes of sheep is shown in Table 1.

The negative control conducted with phosphate-buffered saline (PBS) showed no effect after 24 h, meaning an effectiveness of 0%. In contrast, the positive control using albendazole at 0.25 mg/mL demonstrated a significant effect of 59% at 24 h.

The RP-HPLC-DAD method was used to identify and quantify the rosmarinic acid content of the MeOH extract and *n*-hexane, DCM, EtOAc, *n*-BuOH, and H_2_O subextracts. The amount of rosmarinic acid in the MeOH extract was 0.21 mg/100 mg dry extract. The rosmarinic acid content of the EtOAc extract, which was the most active extract of *N. racemosa,* was determined to be 14.50 mg/100 mg dry extract. *n*-Hexane, DCM, *n*-BuOH, and H_2_O extracts of rosmarinic acid contents were trace amounts and lower than the LOQ value.

**Figure 1:**
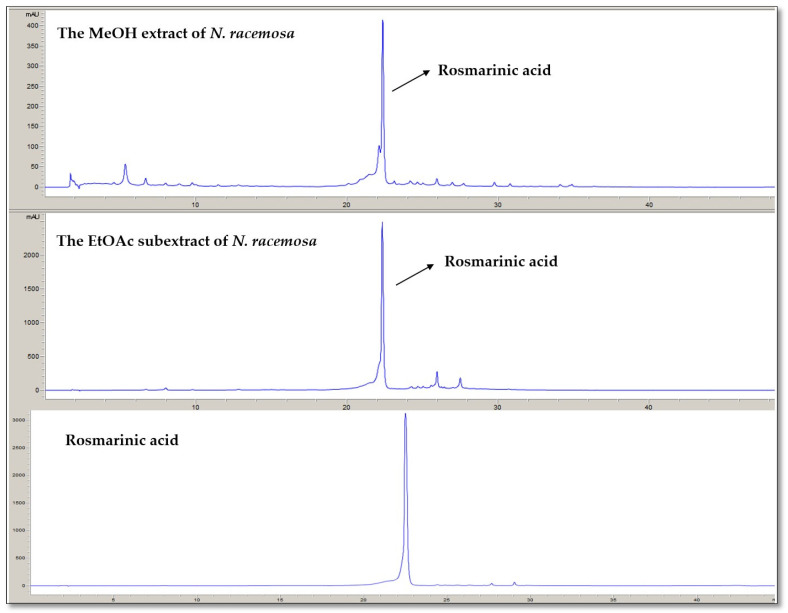
The HPLC chromatograms of the MeOH extract, EtOAc subextract, and rosmarinic acid (330 nm).

Figure 1 shows the HPLC chromatograms of the MeOH extract and EtOAc subextract. Table 2 provides the retention time of rosmarinic acid, test ranges, LOD and LOQ values, and the linear connection between peak area and concentration.

Rosmarinic acid was docked onto the colchicine binding site of the tubulin protein to examine its binding interactions. Rosmarinic acid was selected for in silico studies since the major component of the EtOAc extract was rosmarinic acid. The binding affinity of the compound was found to be −8.1 kcal/mol. Rosmarinic acid showed a similar activity spectrum to the anthelmintic drug albendazole, which binds to the colchicine-binding site of the tubulin protein [15]. As the literature suggests, small molecules like chalchones [16], heterocyclic derivatives [17], and carboxylic acids [18] have a tendency to bind the colchicine-binding site of tubulin. As tubulin plays an essential role in the mechanism of anthelmintic drugs, it may be the main target of rosmarinic acid. Given the data, the colchicine-binding site of the protein was chosen for in silico binding studies.

**Figure 2:**
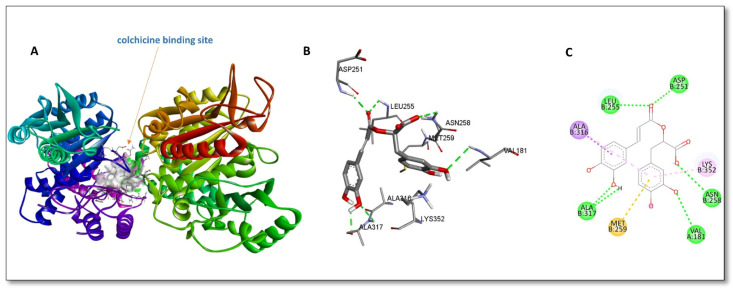
In silico binding interactions between rosmarinic acid and tubulin (PDB code: 5OV7). (**A**) Rosmarinic acid docked on the colchicine-binding site. (**B**) Three-dimensional representation of interactions between rosmarinic acid and colchicine-binding-site residues. (**C**) Two-dimensional representation of interactions between rosmarinic acid and colchicine-binding-site residues. Green, purple, pink, and yellow dashed lines represent hydrogen bonds, pi–sigma bonds, pi–alkyl bonds, and pi–sulphur bonds, respectively.

As shown in Figure 2, rosmarinic acid was highly stabilized by hydrogen bonds on the binding site. Six hydrogen bond interactions were observed between rosmarinic acid and the binding site residues. Two hydrogen bonds were seen between Ala307 and the phenolic hydroxyl of rosmarinic acid. The ester carbonyl of the rosmarinic acid interacted with binding site residues Leu255 and Asp251 through hydrogen bonds. Furthermore, Val181 and Asn258 exhibited hydrogen bonding via one of the phenolic hydroxyls and the oxygen of the carboxylate function, respectively. The compound also stabilized by different interactions like pi–sigma, pi–alkyl, and pi–sulphur interactions provided by amino acid residues Ala316 and Met259.

Research into the genus *Nepeta* consistently reveals a broad spectrum of bioactivities. Bandh et al. (2011) first demonstrated the anthelmintic properties of *Nepeta cataria* extracts against gastrointestinal nematodes in sheep [11]. The significant antiparasitic activity of *Nepeta leavigata* and *Nepeta kurramensis* against *Leishmania* and malaria parasites [19] and *Nepeta menthoides* against *Anopheles stephensi*, a malaria vector [20], has been reported in the literature. Kaska et al. (2018) added new results to the antiparasitic research, demonstrating the efficacy of *Nepeta cadmea* extracts against *Tubifex tubifex*, with potency increasing with concentration [12]. The findings of this study align with the growing amount of research highlighting the anthelmintic properties of the *Nepeta* genus. In addition, the nematicidal activities of *Nepeta cataria* against *Meloidogyne incognita* [21] and essential oils from *Nepeta nuda* ssp. *pubescens* and *Nepeta curviflora* against *Panagrolaimus rigidus* have also been studied [22]. Previous studies have demonstrated the anthelmintic effects of various *Nepeta* species against different helminths [11,12], supporting our observation that *N. racemosa* possesses significant anthelmintic potential. These findings collectively highlight the diverse bioactivities within *Nepeta* species, suggesting their potential as a valuable source of natural products for various applications in pest and parasite control, as well as therapeutic development.

It is commonly known that the class, structure, and concentration of secondary metabolites influence their anthelmintic activity [23]. Furthermore, the actions of these metabolites vary according to the target parasite species and stage of life. According to the authors, these variations may be due to variations in the makeup of particular parasite sheath proteins, which have distinct interactions with the chemical groups [24,25]. Since the structural components of the eggshell and larval coat differ, as has also been shown with traditional anthelmintic medications, the same conclusion can be drawn regarding variations among parasite stages [26]. In general, larval ensheathment inhibition assay IC50 findings are often reported to be lower than those of egg hatching inhibition assays, indicating that infectious L3 larvae are more vulnerable than eggs [27,28].

Rosmarinic acid is a polyphenolic secondary metabolite found in many plants, including plants of the Lamiaceae family. The compound was first isolated from *Salvia rosmarinus* (syn: *Rosmarinus officinalis*) [29]. The addition of plants containing rosmarinic acid to livestock diets has shown encouraging results in improving immunological indices, growth, feed utilization, fertility, antioxidant status, productivity, and reproductive performance [30]. Studies in the literature have suggested that rosmarinic acid may exert anthelmintic effects through mechanisms such as disrupting the metabolic processes of various parasites or interfering with their cellular functions [31,32]. Compared to albendazole, which resulted in an 89.1% decrease in *Aspiculurus tetraptera* counts, rosmarinic acid raised worm load by −8.17% [33]. Many studies have focused on the antioxidant effect of rosmarinic acid. Its high antioxidant properties may indirectly support the anthelmintic effect by increasing the host’s immune response against helminths [34]. Another essential effect of rosmarinic acid is its anti-inflammatory effect, demonstrated by in vitro and in vivo studies on various inflammatory diseases [35]. Inflammation in parasite infections often weakens host tissues and provides suitable conditions for parasite survival [36]. According to the data in the literature, rosmarinic acid contributes to increasing the host’s resistance to infection by reducing oxidative stress and inflammation. In some studies in the literature, the anthelmintic activity of some plants and herbal mixtures containing rosmarinic acid against *H. contortus* was also evaluated [37,38]. However, there is no study in the literature on the anthelmintic effect of rosmarinic acid.

One well-documented target for anthelmintic and anticancer medications is tubulin. Research on tubulin inhibitors may contribute to developing new anthelminthic drugs. Furthermore, tubulin plays a pivotal role in microtubule formation, and the inhibitors have been observed to bind specifically to β-tubulin in nematodes, cestodes, and flukes, leading to structural and functional impairment of microtubules [39,40,41]. All eukaryotes rely on microtubules, which are highly robust and ubiquitous cell organelles, for a range of essential functions, including transport, motility, and mitosis. Many of these designs are in a fragile equilibrium, whereby the collection and disassembly of the dissolvable subunits are regulated. In such instances, the cooperation of drugs and tubulin disrupts this equilibrium, resulting in the complete absence of microtubules and tubulin aggregation [42]. A considerable number of biological functions are also dependent on microtubules. The organism ultimately succumbs to microtubule breakdown [41]. By binding to the β-tubulin colchicine-sensitive region and inhibiting its polymerization and assembly into microtubules, albendazole causes the degeneration of the intestinal cells of worms [43]. Due to its broad-spectrum activity, albendazole is employed as a standard medication in our study. The findings of our study reveal that rosmarinic acid, the primary constituent of the *N. racemosa* plant, exhibits a degree of efficacy that is comparable to that of the anthelmintic drug albendazole.

The LC50 values further support this observation, with the MeOH extract exhibiting the lowest LC50 (5.432), suggesting a higher potency compared to the other extracts. The relatively similar LC50 values for the remaining extracts suggest that, although their efficacies differ in the larval motility assay, their overall potency in terms of the concentration required for 50% mortality is comparable. Conversely, in order to assess the toxicity of these extracts in sheep, it is recommended that in vitro studies on cell cultures be carried out and followed by in vivo studies in sheep, starting under controlled conditions and subsequently progressing to an on-farm study.

Although the highest activity was observed in the EtOAc subextract carrying rosmarinic acid as the major compound, significant activity was also observed in other extracts. This suggests that other phytochemicals carried by these extracts containing trace amounts of rosmarinic acid may have a synergistic effect on rosmarinic acid for anthelminthic activity. Further studies on phytochemicals that may contribute to the anthelmintic activity in other extracts are needed.

## 3. Materials and Methods

### 3.1. Plant Material

The aerial parts of *Nepeta recemosa* Lam. were collected from Tepeköy, Şavşat, Artvin, in the flowering period. Prof. Dr. Hayri Duman identified the plant. A voucher specimen of the plant has been kept in the Herbarium of the Faculty of Pharmacy, Gazi University (Herbarium Number: BK8).

### 3.2. Extraction Procedure

The powdered plant (700 g) was extracted with methanol (MeOH) (Sigma-Aldrich, St. Louis, MO, USA) at room temperature. The extract was dried by evaporating at 40 °C under low pressure. The crude MeOH extract (163.45 g) was subsequently fractionated with *n*-hexane (Sigma-Aldrich, USA), DCM (Supelco, USA), EtOAc (Sigma-Aldrich, USA), and *n*-BuOH (Sigma-Aldrich, USA) in a separatory funnel. Each subextract evaporated to dryness under reduced pressure by a rotary evaporator. The remaining aqueous phase (R-H_2_O) after solvent extractions lyophilized to completely remove water. The yields of the subextract were “*n*-hexane subextract” (13.64%), “DCM subextract” (7.57%), “EtOAc subextract” (3.41%), and “*n*-BuOH subextract” (4.88%), respectively.

### 3.3. Anthelmintic Activity Studies

To determine the anthelmintic effects of the extracts, both the egg hatch assay (EHA) and larval motility inhibition assays were utilized.

#### 3.3.1. Egg Hatch Assay

Fecal samples were collected from a commercially raised sheep herd in Antalya province, naturally infected with gastrointestinal nematodes, to isolate gastrointestinal nematode eggs [44]. The technique for the recovery of nematode eggs from the feces was carried out essentially as described by Coles et al. (1992), resulting in a final concentration of 100–200 eggs per millilitre. After centrifuging the suspension for five minutes at 1500 rpm (Nüve NF 815 centrifuge, Ankara, Türkiye), the supernatant was collected. Each well of a 48-well microtiter plate was filled with approximately 100 eggs suspended in 200 μL of distilled water. Plant extracts were then added to each test well at concentrations of 50, 25, 12.5, 6, 3, and 1.5625 mg/mL, bringing the total volume to 400 μL per well. A negative control was prepared using 200 μL of 99.8% PBS and 5% DMSO. A positive control was established with 200 μL of 99.8% albendazole at a concentration of 0.25 mg/mL. After incubating for 48 h at 27 °C, a drop of Lugol’s iodine solution was added to each well to prevent further hatching. The number of unhatched eggs and L_1_ larvae in each well was counted using an Olympus CX21 microscope, Hamburg, Germany. These counts were then used to calculate the percentage inhibition of egg hatching:

Inhibition (%) = 100 (1 − *P*_test_/*P*_control_)

where *P* = number of eggs hatched in EHA

#### 3.3.2. Preparation and Maturation of Trichostrongylid Larvae

To obtain infective gastrointestinal nematode larvae, one kilogram of feces from naturally infected sheep in Antalya Province was combined with wood shavings in a plastic container. This mixture was incubated at 27 °C for 10–11 days (using a Nüve ES 110 incubator), allowing gastrointestinal nematode eggs to hatch and develop into infective L_3_ larvae. After incubation, the mixture was transferred to a Baermann’s apparatus and left overnight, facilitating larval migration. The L_3_ larvae were collected from the test tubes attached to the apparatus and concentrated by centrifugation (five cycles at 1000 rpm for 5 min). Using a micropipette, the larvae were carefully selected, washed three times with PBS, and stored at 8 °C for future experiments [45].

#### 3.3.3. Larval Motility Assays

Dried plant extracts were dissolved in a solution of distilled water and 5% dimethyl sulfoxide (DMSO). For the modified larval motility assay, 100 larvae were incubated with plant extracts in PBS (pH 7.2; 15–30 °C) at concentrations ranging from 1.5625 to 50 mg/mL, while PBS and 5% DMSO served as a negative control. Albendazole at a concentration of 0.25 mg/mL was used as a positive control. Larval mortality was assessed at 0, 1, 2, 3, 4, 6, 8, and 24 h after incubation. Larval mortality was determined by observing brightness, straightness, and the absence of movement. The experiment was conducted in triplicate for each concentration and time of plant extracts [46]. LC50 value was calculated using the AAT Bioquest Programme [47].

#### 3.3.4. Statistical Analysis

GraphPad Prism Version 6.01 (San Diego, CA, USA) was utilized for statistical analysis. Dunnett’s Post Hoc Test was used to perform a one-way ANOVA on each parameter. The results’ statistical significance was compared with the control group and was expressed as follows:

^a^: *p* < 0.01; ^b^: *p* < 0.001

### 3.4. Quantification of Rosmarinic Acid Using the RP-HPLC-DAD Method

The quantitative analysis of rosmarinic acid (Sigma-Aldrich, USA) in the extract and the subextracts was performed using the RP-HPLC-DAD. After dissolving all extracts in 25% (*v*/*v*) acetonitrile solution to a concentration of 5/10 mg/mL, membrane filters were used to filter them.

Rosmarinic acid was also prepared in a 25% acetonitrile solution at different concentrations. The HP Agilent 1260 series LC System, HP Agilent 1260 4 (quaternary pump) LC Pump and ACE 5 C18 (5 µm, 150 mm × 4.6 mm) column were used. The temperature of the column was 25 °C. The mobile phase used was a mixture of Solvent A (Acetonitrile) and Solvent B (H_2_O: formic acid [100:0.1]). The peaks were separated by using the gradient method with a flow rate of 1 mL/min. After injecting 20 μL of the extract and the standard solutions into the column, the chromatograms were recorded from 330 to 350 nm. The flow program was as follows: 10 min 20% A, to 15 min 85% A, to 35 min 20% A, to 45 min 20% A. Finally, the calibration equation and correlation coefficient for rosmarinic acid were found.

#### Validation

The standard solution of rosmarinic acid was prepared with 6 different concentrations from 10 ppm to 1000 ppm. For the quantitative analysis, the external standard approach was used. The average of the areas under the peaks for each concentration was determined after standard substances were examined three times in HPLC to generate the calibration curve. The extracts were produced at 5/10 mg/mL concentrations. The International Conference on Harmonization (ICH) validation and analytical method Q2 provided the basis for determining the validation parameters [48]. The approach was used to determine the test range, limit of quantitation (LOQ), recovery, and limit of detection (LOD).

### 3.5. In Silico Studies

Molecular docking studies were performed in order to examine the binding interactions and affinities of rosmarinic acid. The docking studies were performed using Chimera 1.17.3. In this study, since the potential target is tubulin, the tubulin structure (PDB code: 5OV7) containing a co-crystalized ligand, rigosertib, was selected to be docked. After ligand preparation and protein preparation steps, the compound was docked onto the colchicine-binding site. The best-scoring docking pose was chosen for further examination. Visualizations of 2D and 3D interactions between rosmarinic acid and binding site residues were performed in Biovia Discovery Studio 21.1 [16].

## 4. Conclusions

In conclusion, the anthelmintic activities of the different *Nepeta racemosa* solvent extracts depending on the concentration were evaluated. The efficacy of the *N. racemosa* EtOAc subextract (NR-EtOAc) showed the highest effectiveness at 79.66%, and the lowest effectiveness was observed with *n*-BuOH at 51.66%. The amount of rosmarinic acid in the extracts was calculated using HPLC, and as a result of this analysis, it was determined that in the EtOAc fraction, which showed the highest activity, it was found to be 14.50 mg/100 mg dry extract. Rosmarinic acid showed a similar activity spectrum to the anthelmintic drug albendazole, which binds to the colchicine-binding site of the tubulin protein in in silico studies. The literature reported that rosmarinic acid has an anthelmintic effect, and this effect can be realized through its antioxidant and anti-inflammatory effects. In light of all these, it is thought that *N. racemosa* may exert its anthelmintic activity via rosmarinic acid. 

The development of anthelmintic resistance in livestock parasites is a major concern globally. The use of plant-derived anthelmintics, with their potential for diverse mechanisms of action compared to synthetic drugs, presents a promising strategy to combat this resistance. The high efficacy of *N. racemosa* extracts in inhibiting egg hatching and killing larvae suggests a potential role in integrated parasite management strategies. However, further research is needed to determine the long-term efficacy and safety of *N. racemosa* extracts in vivo, as well as to elucidate the specific mechanisms of action involved. The remarkable diversity within the *Nepeta* genus, particularly in regions like Türkiye and Iran, warrants further investigation to identify other promising species with anthelmintic potential.

Although the efficacy of rosmarinic acid was evaluated in this study, further studies on other extracts are needed.

## Figures and Tables

**Table 1:** The mortality (%) and LC50 value of plant extracts obtained by different methods on 3rd-stage larvae of gastrointestinal nematodes of sheep.

Treatment Group	Concentration (mg/mL)	Mortality (%) ± S.E.M							LC50 for L_3_ Stages of Gastrointestinal Nematodes of Sheep
		1 h	2 h	3 h	4 h	6 h	8 h	24 h	
NR-MeOH	50	48.0 ± 0.8 ^b^	55.0 ± 0.0 ^b^	68.0 ± 1.4 ^b^	72.0 ± 1.2 ^b^	74.0 ± 0.0 ^b^	74.0 ± 0.0 ^b^	74.0 ± 0.0 ^b^	5.432
	25	41.7 ± 0.6 ^b^	46.3 ± 0.6 ^b^	52.3 ± 0.6 ^b^	65.7 ± 1.2 ^b^	69.3 ± 1.0 ^b^	69.3 ± 1.0 ^b^	69.3 ± 1.0 ^b^	
	12.50	26.0 ± 0.7 ^b^	41.7 ± 0.6 ^b^	48.7 ± 0.6 ^b^	60.7 ± 1.2 ^b^	65.7 ± 0.5 ^b^	65.7 ± 0.5 ^b^	65.7 ± 0.5 ^b^	
	6.25	22.7 ± 0.6 ^b^	39.7 ± 0.2 ^b^	45.7 ± 0.5 ^b^	49.7 ± 1.5 ^b^	57.7 ± 0.5 ^b^	57.7 ± 0.5 ^b^	57.7 ± 0.5 ^b^	
	3.125	14.3 ± 0.5 ^b^	20.7 ± 0.5 ^b^	26.0 ± 0.7 ^b^	27.7 ± 0.3 ^b^	39.7 ± 0.6 ^b^	39.7 ± 0.6 ^b^	39.7 ± 0.6 ^b^	
	1.5625	9.7 ± 0.2 ^b^	13.3 ± 0.0 ^b^	18.7 ± 0.3 ^b^	34.4 ± 1.2 ^b^	34.4 ± 1.2 ^b^	34.4 ± 1.2 ^b^	34.4 ± 1.2 ^b^	
NR-*n*-Hexane	50	40.3 ± 0.6 ^b^	45.3 ± 0.6 ^b^	53.0 ± 1.1 ^b^	64.7 ± 0.9 ^b^	67.0 ± 0.0 ^b^	67.0 ± 0.0 ^b^	67.0 ± 0.0 ^b^	7.3035
	25	35.0 ± 0.4 ^b^	38.3 ± 0.6 ^b^	45.7 ± 0.9 ^b^	60.7 ± 0.7 ^b^	64.0 ± 0.4 ^b^	64.5 ± 0.5 ^b^	64.5 ± 0.5 ^b^	
	12.50	22.0 ± 0.0 ^b^	34.7 ± 0.5 ^b^	40.7 ± 0.5 ^b^	54.7 ± 0.3 ^b^	58.3 ± 0.6 ^b^	58.0 ± 0.0 ^b^	58.0 ± 0.0 ^b^	
	6.25	17.7 ± 0.6 ^b^	26.0 ± 0.7 ^b^	31.0 ± 0.7 ^b^	36.7 ± 0.7 ^b^	42.0 ± 0.8 ^b^	46.0 ± 0.0 ^b^	46.0 ± 0.0 ^b^	
	3.125	10.7 ± 0.5 ^b^	14.7 ± 0.5 ^b^	20.0 ± 0.0 ^b^	23.0 ± 0.6 ^b^	30.7 ± 0.5 ^b^	36.0 ± 0.0 ^b^	36.0 ± 0.0 ^b^	
	1.5625	7.3 ± 0.2 ^a^	10.7 ± 0.5 ^b^	14.0 ± 0.4 ^b^	15.7 ± 0.3 ^b^	26.7 ± 0.6 ^b^	30.0 ± 0.8 ^b^	30.0 ± 0.8 ^b^	
NR-DCM	50	32.7 ± 0.5 ^b^	40.0 ± 0.8 ^b^	45.7 ± 0.6 ^b^	53.7 ± 0.9 ^b^	58.7 ± 0.6 ^b^	61.0 ± 1.1 ^b^	61.0 ± 1.1 ^b^	9.8452
	25	23.7 ± 0.6 ^b^	33.7 ± 0.6 ^b^	41.7 ± 0.6 ^b^	46.3 ± 0.9 ^b^	51.0 ± 0.7 ^b^	58.0 ± 1.1 ^b^	58.0 ± 1.1 ^b^	
	12.50	16.7 ± 0.9 ^b^	26.0 ± 0.8 ^b^	36.7 ± 0.5 ^b^	45.7 ± 1.2 ^b^	50.7 ± 0.8 ^b^	50.7 ± 0.8 ^b^	50.7 ± 0.8 ^b^	
	6.25	12.3 ± 0.8 ^b^	16.0 ± 0.7 ^b^	22.7 ± 1.0 ^b^	27.0 ± 0.6 ^b^	40.7 ± 1.2 ^b^	40.7 ± 1.2 ^b^	40.7 ± 1.2 ^b^	
	3.125	14.4 ± 0.9 ^b^	16.0 ± 0.4 ^b^	17.0 ± 0.4 ^b^	21.3 ± 1.2 ^b^	32.0 ± 0.8 ^b^	32.0 ± 0.8 ^b^	32.0 ± 0.8 ^b^	
	1.5625	11.0 ± 0.7 ^b^	15.0 ± 0.0 ^b^	17.0 ± 0.7 ^b^	19.7 ± 0.9 ^b^	23.3 ± 0.6 ^b^	28.0 ± 0.8 ^b^	28.0 ± 0.8 ^b^	
NR-EtOAc	50	55.3 ± 1.2 ^b^	65.0 ± 0.7 ^b^	72.3 ± 1.0 ^b^	75.0 ± 1.2 ^b^	79.7 ± 1.4 ^b^	79.7 ± 1.4 ^b^	79.7 ± 1.4 ^b^	7.5025
	25	50.3 ± 0.8 ^b^	57.0 ± 0.8 ^b^	63.3 ± 0.6 ^b^	69.7 ± 0.9 ^b^	72.3 ± 0.8 ^b^	72.3 ± 0.8 ^b^	72.3 ± 0.8 ^b^	
	12.50	48.3 ± 0.8 ^b^	54.3 ± 0.8 ^b^	56.0 ± 1.1 ^b^	62.0 ± 2.1 ^b^	62.7 ± 1.2 ^b^	66.3 ± 0.8 ^b^	66.3 ± 0.8 ^b^	
	6.25	44.3 ± 0.2 ^b^	46.7 ± 0.6 ^b^	50.7 ± 0.8 ^b^	56.0 ± 1.0 ^b^	61.0 ± 1.1 ^b^	61.0 ± 1.1 ^b^	61.0 ± 1.1 ^b^	
	3.125	28.0 ± 0.4 ^b^	32.3 ± 0.8 ^b^	38.0 ± 1.1 ^b^	45.7 ± 0.7 ^b^	50.7 ± 0.8 ^b^	50.7 ± 0.8 ^b^	50.7 ± 0.8 ^b^	
	1.5625	24.7 ± 0.2 ^b^	26.7 ± 0.8 ^b^	33.3 ± 0.8 ^b^	39.7 ± 1.2 ^b^	45.3 ± 0.6 ^b^	45.3 ± 0.6 ^b^	45.3 ± 0.6 ^b^	
NR-*n*-BuOH	50	20.0 ± 0.0 ^b^	23.3 ± 0.5 ^b^	31.7 ± 0.8 ^b^	46.7 ± 0.9 ^b^	51.7 ± 0.6 ^b^	51.7 ± 0.6 ^b^	51.7 ± 0.6 ^b^	7.8801
	25	18.0 ± 0.4 ^b^	20.7 ± 0.6 ^b^	27.0 ± 0.4 ^b^	41.3 ± 0.9 ^b^	46.0 ± 0.7 ^b^	46.0 ± 0.7 ^b^	46.0 ± 0.7 ^b^	
	12.50	17.3 ± 0.5 ^b^	19.3 ± 0.2 ^b^	23.7 ± 0.8 ^b^	39.0 ± 1.0 ^b^	39.7 ± 0.8 ^b^	39.7 ± 0.8 ^b^	39.7 ± 0.8 ^b^	
	6.25	15.0 ± 0.4 ^b^	18.0 ± 0.4 ^b^	19.7 ± 0.2 ^b^	29.7 ± 0.9 ^b^	33.0 ± 0.4 ^b^	33.0 ± 0.4 ^b^	33.0 ± 0.4 ^b^	
	3.125	12.7 ± 0.2 ^b^	12.0 ± 0.0 ^b^	16.0 ± 0.7 ^b^	23.0 ± 1.2 ^b^	26.3 ± 0.6 ^b^	26.3 ± 0.6 ^b^	26.3 ± 0.6 ^b^	
	1.5625	8.0 ± 0.0 ^b^	9.7 ± 0.5 ^b^	10.3 ± 0.2 ^b^	16.3 ± 0.3 ^b^	21.0 ± 0.7 ^b^	21.0 ± 0.7 ^b^	21.0 ± 0.7 ^b^	
NR-H_2_O	50	26.0 ± 0.7 ^b^	34.3 ± 0.8 ^b^	47.0 ± 0.8 ^b^	54.7 ± 0.9 ^b^	64.0 ± 0.7 ^b^	64.5 ± 0.3 ^b^	64.0 ± 0.7 ^b^	6.8703
	25	22.0 ± 0.4 ^b^	30.7 ± 0.8 ^b^	41.7 ± 0.8 ^b^	51.3 ± 0.9 ^b^	59.0 ± 0.4 ^b^	59.0 ± 0.4 ^b^	59.0 ± 0.4 ^b^	
	12.50	17.3 ± 0.6 ^b^	26.3 ± 0.6 ^b^	34.0 ± 1.1 ^b^	45.0 ± 0.6 ^b^	52.7 ± 1.5 ^b^	52.7 ± 1.5 ^b^	52.7 ± 1.5 ^b^	
	6.25	14.3 ± 0.2 ^b^	20.3 ± 0.2 ^b^	26.7 ± 0.5 ^b^	32.0 ± 1.2 ^b^	40.3 ± 1.0 ^b^	40.3 ± 1.0 ^b^	40.3 ± 1.0 ^b^	
	3.125	12.0 ± 0.0 ^b^	15.0 ± 0.0 ^b^	18.0 ± 0.4 ^b^	22.0 ± 1.5 ^b^	30.0 ± 0.0 ^b^	30.0 ± 0.0 ^b^	30.0 ± 0.0 ^b^	
	1.5625	9.0 ± 0.4 ^b^	10.7 ± 0.5 ^b^	13.0 ± 0.0 ^b^	16.0 ± 0.0 ^b^	18.7 ± 0.6 ^b^	18.7 ± 0.6 ^b^	18.7 ± 0.6 ^b^	
Albendazole	0.25	23.0 ± 0.0 ^b^	30.0 ± 0.0 ^b^	42.0 ± 0.0 ^b^	52.0 ± 0.0 ^b^	59.0 ± 1.0 ^b^	59.0 ± 1.0 ^b^	59.0 ± 1.0 ^b^	
PBS	0	0	0	0	0	0	0	0	-
DSMO	0	0	0	0	0	0	0	0	-

NR-MeOH: *N. racemosa* methanol extract; NR-*n*-hexane: *N. racemosa n*-hexane extract; NR-DCM: *N. racemosa* dichloromethane extract; NR-EtOAc: *N. racemosa* ethyl acetate extract; NR-*n*-BuOH: *N. racemosa n*-butanol extract; NR-H_2_O: *N. racemosa* aqueous extract; LC50: lethal concentration 50; PBS: phosphate-buffered saline; ^a^: *p* < 0.01; ^b^: *p* < 0.001; S.E.M: standard error of the mean.

**Table 2:** Retention time, linear relationships between peak areas and concentrations, test ranges, LOD and LOQ of rosmarinic acid.

Compound	Retention Time (min)	Standard Curve	R^2^	Test Range (µg/mL)	LOD (µg/mL)	LOQ (µg/mL)
Rosmarinic acid	22.441	y = 69.095x + 2194.4	0.9939	10–1000	0.6198	1.8782

y: peak area; x: concentration (mg/mL); LOD = limit of detection 3.3× SD/m; LOQ = limit of quantification 10× SD/m.

## Data Availability

Raw data of this study are available upon request from the corresponding author.

## References

[1] Štrbac F., Bosco A., Maurelli M.P., Ratajac R., Stojanović D., Simin N., Orčić D., Pušić I., Krnjajić S., Sotiraki S. (2022). Anthelmintic Properties of Essential Oils to Control Gastrointestinal Nematodes in Sheep—In Vitro and In Vivo Studies. Vet. Sci..

[2] Beleckė A., Kupčinskas T., Stadalienė I., Höglund J., Thamsborg S.M., Stuen S., Petkevičius S. (2021). Anthelmintic resistance in small ruminants in the Nordic-Baltic region. Acta Vet. Scand..

[3] Kozan E., Tatli I.I., Kahraman C., Kupeli Akkol E., Akdemir Z. (2011). The in vivo Anthelmintic Efficacy of some *Verbascum* species growing in Turkey. Exp. Parasitol..

[4] Charlier J., Rinaldi L., Musella V., Ploeger H.W., Chartier C., Vineer H.R., Claerebout E. (2020). Initial assessment of the economic burden of major parasitic helminth infections to the ruminant livestock industry in Europe. Prev. Vet. Med..

[5] Zeineldin M., Abdelmegeid M., Barakat R., Ghanem M. (2018). A review: Herbal medicine as an effective therapeutic approach for treating digestive disorders in small ruminants. Alex. J. Vet. Sci..

[6] Erez M.S., Kozan E. (2018). Anthelmintic resistance in farm animals. Kocatepe Vet. J..

[7] Dag S., Erez M., Kozan E., Gençler Özkan A.M., Çankaya İ. (2023). In vitro anthelmintic activity of five different *Artemisia* L. species growing in Türkiye. Pak. Vet. J..

[8] Sharma A., Cooper R., Bhardwaj G., Cannoo D.S. (2021). The genus *Nepeta*: Traditional uses, phytochemicals and pharmacological properties. J. Ethnopharmacol..

[9] Zengin G., Mahomoodally M.F., Aktumsek A., Jekő J., Cziáky Z., Rodrigues M.J., Custodio L., Polat R., Cakilcioglu U., Ayna A. (2021). Chemical Profiling and Biological Evaluation of *Nepeta baytopii* Extracts and Essential Oil: An Endemic Plant from Turkey. Plants.

[10] Acquaviva A., Di Simone S.C., Nilofar, Bouyahya A., Zengin G., Recinella L., Leone S., Brunetti L., Uba A.I., Guler O. (2023). Screening for Chemical Characterization and Pharmacological Properties of Different Extracts from *Nepeta italica*. Plants.

[11] Bandh S.A., Lone B., Chishti M.Z., Kamili A.N., Ganai B.A., Saleem S. (2011). Evaluation of Anthelmintic and Antimicrobial Activity of the Methanolic Extracts of *Nepeta cataria*. N.Y. Sci. J..

[12] Kaska A., Deniz N., Çiçek M., Mammadov R. (2018). Evaluation of Antioxidant Properties, Phenolic Compounds, Anthelmintic, and Cytotoxic Activities of Various Extracts Isolated from *Nepeta cadmea*: An Endemic Plant for Turkey. J. Food Sci..

[13] Fennell B., Naughton J., Barlow J., Brennan G., Fairweather I., Hoey E., McFerran N., Trudgett A., Bell A. (2008). Microtubules as antiparasitic drug targets. Expert. Opin. Drug Discov..

[14] Ranjan P., Kumar S.P., Kari V., Jha P.C. (2017). Exploration of interaction zones of β-tubulin colchicine binding domain of helminths and binding mechanism of anthelmintics. Comput. Biol. Chem..

[15] Oliva M.Á., Tosat-Bitrián C., Barrado-Gil L., Bonato F., Galindo I., Garaigorta U., Álvarez-Bernad B., París-Ogáyar R., Lucena-Agell D., Giménez-Abián J.F. (2022). Effect of Clinically Used Microtubule Targeting Drugs on Viral Infection and Transport Function. Int. J. Mol. Sci..

[16] Canela M.D., Noppen S., Bueno O., Prota A.E., Bargsten K., Sáez-Calvo G., Jimeno M.L., Benkheil M., Ribatti D., Velázquez S. (2017). Antivascular and antitumor properties of the tubulin-binding chalcone TUB091. Oncotarget.

[17] de la Roche N.M., Mühlethaler T., Di Martino R.M.C., Ortega J.A., Gioia D., Roy B., Prota A.E., Steinmetz M.O., Cavalli A. (2022). Novel fragment-derived colchicine-site binders as microtubule-destabilizing agents. Eur. J. Med. Chem..

[18] Jost M., Chen Y., Gilbert L.A., Horlbeck M.A., Krenning L., Menchon G., Rai A., Cho M.Y., Stern J.J., Prota A.E. (2017). Combined CRISPRi/a-Based Chemical Genetic Screens Reveal that Rigosertib Is a Microtubule-Destabilizing Agent. Mol. Cell.

[19] Memoona A., Amir M.K., Sultan A., Razia P., Sumera P., Lal M., Nisar A. (2012). Evaluation of *Nepeta laevigata*, *Nepeta kurramensis* and *Rhynchosia reniformis* on antimalarial and antileishmanial activities. Int. J. Bioassays.

[20] Mahnaz K., Alireza F., Hassan V., Mahdi S., Reza A.M., Abbas H. (2012). Larvicidal activity of essential oil and methanol extract of *Nepeta menthoides* against malaria vector Anopheles stephensi. Asian Pac. J. Trop. Med..

[21] Pavaraj M., Bakavathiappan G.A., Baskaran S. (2012). Evaluation of some plant extracts for their nematicidal properties against root-knot nematode, *Meloidogyne incognita*. J. Biopest..

[22] Musso L., Scaglia B., Haj G.A., Arnold N.A., Adani F., Scarì G., Dallavalle S., Iriti M. (2017). Chemical characterization and nematicidal activity of the essential oil of *Nepeta nuda* L. ssp. *pubescens* and *Nepeta curviflora* Boiss. from Lebanon. J. Essent. Oil Bear. Plants.

[23] Hoste H., Torres-Acosta J.F., Sandoval-Castro C.A., Mueller-Harvey I., Sotiraki S., Louvandini H., Thamsborg S.M., Terrill T.H. (2015). Tannin containing legumes as a model for nutraceuticals against digestive parasites in livestock. Vet. Parasitol..

[24] Brunet S., Hoste H. (2006). Monomers of condensed tannins affect the larval exsheathment of parasitic nematodes of ruminants. J. Agric. Food Chem..

[25] Quijada J., Fryganas C., Ropiak H.M., Ramsay A., Mueller-Harvey I., Hoste H. (2015). Anthelmintic activities against *Haemonchus contortus* or *Trichostrongylus colubriformis* from small ruminants are influenced by structural features of condensed tannins. J. Agric. Food Chem..

[26] Mansfield L.S., Gamble H.R., Fetterer R.H. (1992). Characterization of the eggshell of *Haemonchus contortus*—I. Structural components. Comp. Biochem. Physiol. B.

[27] Oliveira A.F., Costa Júnior L.M., Lima A.S., Silva C.R., Ribeiro M.N.S., Mesquista J.W.C., Rocha C.Q., Tangerina M.M.P., Vilegas W. (2017). Anthelmintic activity of plant extracts from Brazilian savana. Vet. Parasitol..

[28] Zabré G., Kaboré A., Bayala B., Katiki L.M., Costa-Júnior L.M., Tamboura H.H., Belem A., Abdalla A.L., Niderkorn V., Hoste H. (2017). Comparison of the in vitro anthelmintic effects of *Acacia nilotica* and *Acacia raddiana*. Parasite.

[29] Guan H., Luo W., Bao B., Cao Y., Cheng F., Yu S., Fan Q., Zhang L., Wu Q., Shan M. (2022). A Comprehensive Review of Rosmarinic Acid: From Phytochemistry to Pharmacology and its New Insight. Molecules.

[30] Alagawany M., Abd El-Hack M.E., Farag M.R., Gopi M., Karthik K., Malik Y.S., Dhama K. (2017). Rosmarinic acid: Modes of action, medicinal values and health benefits. Anim. Health Res. Rev..

[31] Wang J., Pan X., Han Y., Guo D., Guo Q., Li R. (2012). Rosmarinic acid from eelgrass shows nematicidal and antibacterial activities against pine wood nematode and its carrying bacteria. Mar. Drugs.

[32] Pinto N.B., Castro L.M., Azambuja R.H.M., Capella G.A., Moura M.Q., Terto W.D., Freitag R.A., Jeske S.T., Villela M.M., Cleff M.B. (2019). Ovicidal and larvicidal potential of Rosmarinus officinalis to control gastrointestinal nematodes of sheep. Brazilian J. Vet. Parasitol..

[33] Eylek B. (2021). Investigating the Anthelmintic Effects of *Rosmarinus officinalis* L. and Rosmarinic Acid in Mice Naturally İnfected with *Aspiculuris tetraptera*. Master’s Thesis.

[34] Adomako-Bonsu A.G., Chan S.L., Pratten M., Fry J.R. (2017). Antioxidant activity of rosmarinic acid and its principal metabolites in chemical and cellular systems: Importance of physico-chemical characteristics. Toxicol. Vitr..

[35] Luo C., Zou L., Sun H., Peng J., Gao C., Bao L., Ji R., Jin Y., Sun S. (2020). A Review of the Anti-Inflammatory Effects of Rosmarinic Acid on Inflammatory Diseases. Front. Pharmacol..

[36] Wang L.J., Cao Y., Shi H.N. (2008). Helminth infections and intestinal inflammation. World J. Gastroenterol..

[37] Seyfried M., Soldera-Silva A., Campestrini L.H., Siebert D.A., Vitali L., Micke G.A., Zawadzki-Baggio S.F., Molento M.B., Maurer J.B.B. (2022). In vitro anthelmintic activity of *Polygonum acre* (Linnaeus, 1754) extracts against the ruminant nematode Haemonchus contortus (Rudolphi, 1803). Arch. Vet. Sci..

[38] Váradyová Z., Mravčáková D., Babják M., Bryszak M., Grešáková L., Čobanová K., Kišidayová S., Plachá I., Königová A., Cieslak A. (2018). Effects of herbal nutraceuticals and/or zinc against *Haemonchus contortus* in lambs experimentally infected. BMC Vet. Res..

[39] Friedman P.A., Platzer E.G. (1978). Interaction of anthelmintic benzimidazoles and benzimidazole derivatives with bovine brain tubulin. Biochim. Biophys. Acta BBA.

[40] Kohler P., Bachmann R. (1981). Intestinal tubulin as possible target for the chemotherapeutic action of mebendazole in parasitic nematodes. Mol. Biochem. Parasitol..

[41] Lacey E. (1988). The role of the cytoskeletal protein, tubulin, in the mode of action and mechanism of drug resistance to benzimidazoles. Int. J. Parasitol..

[42] Kabir M.S.H., Paul A., Mojumdar M., Hasanat A., Islam M., Nabila A., Islam J., Hossain R., Chakrabarty N., Islam N.M. (2016). *In vitro* anthelmintic activity of *Macaranga denticulata* and in silico molecular docking analysis of its isolated compounds with tubulin. Int. J. Pharm..

[43] Horton J. (2000). Albendazole: A review of anthelmintic efficacy and safety in humans. Parasitology.

[44] Coles G.C., Bauer C., Borgsteede F.H.M., Geerts S., Klei T.R., Taylor M.A., Waller P.J. (1992). World Association for the Advancement of Veterinary Parasitology (WAAVP) methods for the detection of anthelmintic resistance in nematodes of veterinary importance. Vet. Parasitol..

[45] Molan A.L., Meagher L.P., Spencer P.A., Sivakumaran S. (2003). Effect of flavan-3-ols on in vitro egg hatching, larval development and viability of infective larvae of *Trichostrongylus colubriformis*. Int. J. Parasitol..

[46] Kotze A.C., Clifford S., O’grady J., Behnke J.M., McCarthy J.S. (2004). An in vitro larval motility assay to determine anthelmintic sensitivity for human hookworm and *Strongyloides* species. Am. J. Trop. Med. Hyg..

[47] AAT Bioquest, Inc Quest Graph™ LC50 Calculator. AAT Bioquest. https://www.aatbio.com/tools/lc50-calculator.

[48] ICH (2022). ICH Guideline Q2(R2), Validation of Analytical Procedures.

